# Adding *ReputationRank* to member promotion using skyline operator in social networks

**DOI:** 10.1186/s40649-018-0055-9

**Published:** 2018-09-04

**Authors:** Jiping Zheng, Siman Zhang

**Affiliations:** 1 College of Computer Science and Technology, Nanjing University of Aeronautics and Astronautics, Nanjing, China; 2 Collaborative Innovation Center of Novel Software Technology and Industrialization, Nanjing, China

**Keywords:** Social networks, Member promotion, *ReputationRank*, Skyline distance, Infra-skyline

## Abstract

**Background:**

To identify potential stars in social networks, the idea of combining member promotion with skyline operator attracts people’s attention. Some algorithms have been proposed to deal with this problem so far, such as skyline boundary algorithms in unequal-weighted social networks.

**Methods:**

We propose an improved member promotion algorithm by presenting *ReputationRank* based on eigenvectors as well as *Influence* and *Activeness* and introduce the concept of skyline distance. Furthermore, we perform skyline operator over non-skyline set and choose the infra-skyline as our candidate set. The added *ReputationRank* helps a lot to describe the importance of a member while the skyline distance assists us to obtain the necessary condition for not being dominated so that some meaningless plans can be pruned.

**Results:**

Experiments on the DBLP and WikiVote datasets verify the effectiveness and efficiency of our proposed algorithm.

**Conclusions:**

Treating the infra-skyline set as candidate set reduces the number of candidates. The pruning strategies based on dominance and promotion cost decrease the searching space.

## Background

Nowadays, more and more social activities take place in social networks (SNs for short) as the SNs become prevailing, such as sharing information, making friends or finishing some team work with others online. Human behaviours in SNs attract more attentions. We can conclude that different members play different roles, some members may be “leaders” [1], and others who seem ordinary for the moment but it may be outstanding in the future.

To specify who are about to be important in the future, making a standard of importance should be crucial. There are multiple disciplines to recognize an important one. For example, in an online community as “Sina Weibo”, we consider the one who owns lots of followers as important or whose posts get many retweets as important [2]. In a word, different criteria make different “leaders”, the one who does not match the criteria would fail to be important. Usually, a single attribute does not describe the importance of a member accurately. Thus, it is necessary for us to formulate a multi-criteria standard to measure importance. The skyline operator has thus been introduced to do this in SNs. It is well known that the skyline operator is a good tool for multi-criteria decision making. It can be used to query for those objects that are not worse than any other. When the skyline operator was first used to do promoting in SNs, Peng et al. [3] proposed the definition of member promotion and provided the brute-force algorithm to realize it. However, this algorithm was inadvisable for a waste of time and space. Thus the authors introduced the skyline operator and proposed the dominance-based pruning strategy to optimize the ways of result validation. Afterwards, they carried further research on it and put forward the concept of promotion boundary for limiting the promotion plans, thus led to the boundary-based pruning strategy [4]. At the same time, they also proposed a cost-based pruning strategy, which greatly improved the efficiency of member promotion. Nevertheless, the final result was unsatisfactory on account of the simple metric of importance.

In this paper, we mainly study directed social graphs with the knowledge of graph theory [4], taking *Influence*, *Activeness* and *ReputationRank* as metrics of member’s importance. The attributes *Influence* and *Activeness* are easy to understand, and they are indegree and outdegree in a directed graph correspondingly. We consider that if a person owns lots of followers, s/he is influential and if a person follows lots of people, which indicates the ability to reach many other members, s/he is active. What is more, we learn from the idea of Google’s *pagerank* algorithm, a way of measuring the importance of website pages, put forward *ReputationRank* to measure the importance of a member in SNs. Our goal is to find those members who can be “stars” in the future accurately and efficiently. To ensure accuracy, we assume that if a person is followed by some important persons, s/he is important too. Further, we assume that any two members in a specific direction can be connected only once and we employ edge addition as the promotion manner to simulate the process of relationship established. Usually, it will take cost to add new edges between two nodes. Therefore, the problem of member promotion in SNs is defined to excavate the most appropriate non-skyline member(s) which can be promoted to be skyline member(s) by adding new edges with the minimum cost. However, the calculation of added *ReputationRank* metric involves series of mathematical operations, it may need enormous computational cost.

To ensure efficiency and tackle the challenge of the computation cost, we mainly consider the changes of *Influence* and *Activeness* after adding edges, because we only need to add the number of directed edges involved. However, when calculating a point’s *ReputationRank*, it involves some complicated matrix operations. We need to take the total number of the members as denominator. Apparently, for the great changes of the denominator (we assume the SN is dynamic), the subtle changes of numerator can be ignored. We conduct a skyline query on the dimensions of *Influence*, *Activeness* and *ReputationRank* to get the non-skyline set, then we carry out a second skyline query on the non-skyline set. We treat the skyline set in the second skyline query as our candidate set. It helps to reduce the number of candidates greatly. The contributions of this paper are summarized as follows.

- We learn from the *pagerank* algorithm and propose to add the *ReputationRank* to measure the importance of a member, which helps to improve the accuracy of the prediction.
- We carry a second skyline query over the non-skyline set which is obtained from the skyline query on the three-dimensional dataset and regard the infra-skyline as our candidates. It remarkably reduces the number of candidates. Then we introduce the skyline distance and the cost-based as well as dominance-based strategies to prune some meaningless promotion plans.
- Experiments on DBLP and WikiVote datasets are conducted to show the effectiveness and efficiency of our approach.

The rest of this paper is organized as follows. “Related work” section reviews related work. In “Preliminaries” section, we introduce several preliminary concepts. Then we bring forward the problem and propose the algorithm with analysis in “Prediction of promoting members in SNs” section. The results of the experiments are presented to show the effectiveness and efficiency of our algorithm in “Experimental analysis” section. Finally, we conclude our work in “Conclusions” section.

## Related work

### Skyline queries

The skyline operator was first introduced by Börzsöny et al. [5]. It was a tool for multi-criteria decision making. Then some representative algorithms for skyline computation were proposed, such as Block-Nested-Loops (BNL) and Divide-and-Conquer (D&C) [5], Bitmap and Index [6], Nearest Neighbor (NN) [7], and the Branch and Bound Skyline (BBS) algorithm [8]. Both BNL and D&C had to traverse the entire dataset before returning skyline points. The bitmap-based method transformed each data points to bit vectors. In each dimension, the value was represented by the same number ‘1’. However, it could not guarantee a good initial response time and the bitmaps would be very large for large values. Therefore, another method which transformed multiple dimensions into a single one space where objects were clustered and indexed using a $B^{+}$ tree was raised. It helped a lot to save processing time because skyline points could be determined without examining the rest of the objects not accessed yet. The NN algorithm was proposed by Kossmann et al. [7]. It could progressively report the skyline set in an order according to user’s preferences. However, one data point may be accessed many times until being dominated. To find remedy for this drawback, Papadias et al. [8] proposed BBS, an *R*-tree based algorithm, which retrieved skyline points by traversing the *R*-tree by the Best-First strategy. There are also lots of studies on skyline variations for different applications such as subspace skylines [9], *k*-dominant skylines [10], probabilistic skyline computation on uncertain data [11], weighted attributes skylines [12], skyline queries over data streams [13], skyline analysis on time series data [14], spatial skyline queries [15], skyline computation in partially ordered domains [16] and using skylines to mine user preferences, making recommendations [17] and searching star scientists [18].

### Member promotion

Peng et al. [3] first proposed the concept of member promotion in SNs and provided a brute-force algorithm to solve it. It stated that member promotion aimed at promoting the unimportant member which was most potential to be promoted and became important one. It considered “most potential” as the minimum promotion cost, which meant the member could be able to be promoted at minimum cost. And the brute-force algorithm tried out all the available added edges to find out the optimal promotion plans. However, some “meaningless” added edges would also be verified, it led to high time cost. Based on the characteristics of the promotion process, Peng et al. [3] proposed the IDP (Index-based Dynamic Pruning) algorithm, which could generate some prunable plans when met a failed promotion plan. Later, Peng et al. [4] conducted further research on the member promotion, which mainly focused on unequal SNs. They brought forward promotion boundary to limit promotion plans. At the same time, they proposed the cost-based and dominance-based pruning strategies to reduce the searching space. Furthermore, the authors expanded the algorithm, proposed an InfraSky algorithm based on equal-weighed SNs. They optimized the cost model and put forward a new concept named “Infra-Skyline” to remarkably prune the candidate space [4]. However, all the works of Peng et al. [3, 4] are limited for only metrics such as indegree and outdegree which could not describe a member’s importance entirely, thus the prediction results of member promotion were not very satisfying.

A major distinction between our approach and Peng et al.’s works is that we add *ReputationRank* as a metric attribute, which is more suitable to describe a member’s characteristic besides the two metrics. With an upgrade of the metrics, our work shows more efficiency.

## Preliminaries

In this paper, SN is modeled as a weighted directed graph *G*(*V*, *E*, *W*). The nodes in *V* represent the members in the SN. Those elements of *E* are the existing directed edges between the members. Each $w\in W$ denotes the cost for establishing the directed edge between any two different members.

### **Definition 1**

(*Influence*) Given a node *v* in an SN *G*(*V*, *E*, *W*), the *Influence* of *v*, marked as *I*(*v*), is the indegree of *v*.

### **Definition 2**

(*Activeness*) Given a node *v* in an SN *G*(*V*, *E*, *W*), the *Activeness* of *v*, marked as *A*(*v*), is the outdegree of *v*.

### **Definition 3**

(*ReputationRank*) Given a node *v* in an SN *G*(*V*, *E*, *W*), the *ReputationRank* of *v*, marked as *P*(*v*), is the value of the corresponding component in the eigenvector of the normalized social relationship matrix whose eigenvalue is 1.

### *Example 1*

Suppose that there are three nodes in an SN, let the nodes be $v_{1}$, $v_{2}$, $v_{3}$, if the SN’s normalized social relationship matrix has an eigenvalue 1 and its corresponding eigenvector is $p=(p_{1}, p_{2}, p_{3})$ (we can obtain these values by the method introduced in “ReputationRank” section), then we know that $v_{1}$, $v_{2}$, $v_{3}$’s *ReputaionRank* is $p_{1}$, $p_{2}$ and $p_{3}$, respectively.

### **Definition 4**

(*Social relationship matrix*) Given an SN *G*(*V*, *E*, *W*), the social relationship matrix is an adjacency matrix which expresses the links between the members in the SN, denoted as *M*.

### **Definition 5**

(*Normalization social matrix*) If a social relationship matrix is *M*, then its normalization social matrix is a matrix where the sum of the elements for each column is 1. We denote the normalization matrix as $M'$.

### **Definition 6**

(*Dominance*) Given an SN *G*(*V*, *E*, *W*), $\forall v_{1}, v_{2} \in V$, we say $v_{1}$ dominates $v_{2}$ if and only if $v_{1}$ is not worse in *Influence* dimension, *Activeness* dimension and *ReputationRank* dimension, and is better in at least one dimension than $v_{2}$.

### **Definition 7**

(*Dominator set*) Given an SN *G*(*V*, *E*, *W*), if $v_{1}$ dominates $v_{2}$, we say $v_{1}$ is a dominator of $v_{2}$. Correspondingly, all dominators of a member *v*, marked as $\delta (v)$, are denoted as the dominator set of *v*.

### **Definition 8**

(*Skyline*) Given an SN *G*(*V*, *E*, *W*), the skyline of *G*, denoted as $S_{G}$, is the set of members which are not dominated by any other member.

### **Definition 9**

(*Infra-skyline*) Given an SN *G*(*V*, *E*, *W*), the infra-skyline of *G* is the skyline of the set of all non-skyline members of *G*, namely, if $S_{G}$ is the skyline set of *G*, then the infra-skyline of *G* is $S_{G-S_{G}}$.

### *Example 2*

Given an SN consists of seven members, namely $\{A, B, C, D, E, F, G\}$, suppose that the skyline set is $\{A, B, D\}$, what is more, *E* is dominated by *F*, then the infra-skyline in the SN is $\{C, F, G\}$.

### **Definition 10**

(*Promotion cost*) Given an SN *G*(*V*, *E*, *W*), the promotion cost of a candidate *c*, is the sum of all the weights corresponding to the edges being added at *c*, denoted as $cost(c, c')=\sum _{e\in E_{a}}\gamma (e)$, where $c'$ is the point after the edges are added at point *c*, $E_{a}$ is the set of added edges and $\gamma (e)$ is the cost of adding edge *e*.

Assume *I*(*v*), *A*(*v*) and *P*(*v*) represent the *Influence*, *Activeness* and *ReputationRank* of node *v* in *V*, respectively. We consider the larger the values of *I*(*v*), *A*(*v*) and *P*(*v*) are, the better they are.

### ReputationRank

*ReputationRank* is obtained by counting the number and quality of followers to a person to determine a rough estimate of how important the person is. The *ReputationRank* of a member is defined recursively and depends on the number and *ReputationRank* metric of all followers. A member that is followed by many members with high *ReputationRank* receives a high rank itself.

From the point of mathematics, members’ *ReputationRank* depends on the reputation of those members who follow them. The *ReputationRank* of the follower also depends on persons who follow her/him, and the subsequent process can be implemented in the same manner. Thus, for solving this kind of “infinite regression”, we define $P(v_{i})$ as the *ReputationRank* of member *i*, and we notice that the *i*th column of the social relationship matrix shows those members who follow her/him. Therefore, we can get $v_{i}$’s *ReputationRank* by adding these products between the relation state and the *ReputationRank* of all other members, namely

1 $$\begin{aligned} P(v_{i})=x_{1i}P(v_{1})+x_{2i}P(v_{2})+\cdots +x_{gi}P(v_{g}), \end{aligned}$$

where the coefficient $x_{ji}$ denotes the reciprocal of outdegree of member *j*, *g* is the number of the members.

#### *Example 3*

**Fig. 1:**
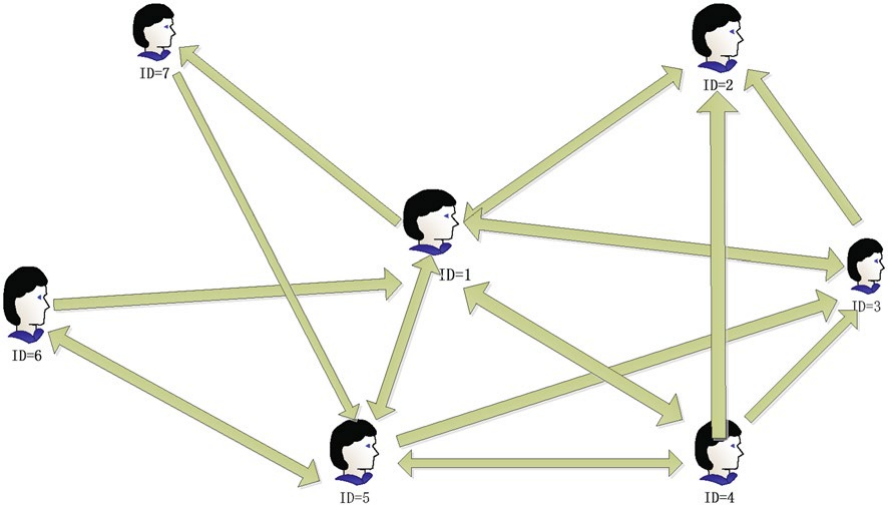
A social network example

If there are seven members in an SN, as shown in Fig. 1, the member $v_{2}$ is followed by $v_{1}$, $v_{3}$ and $v_{4}$, then the rest entries of the second column in the social relationship matrix are all 0s. Furthermore, $v_{1}$’s outdegree is 5, $v_{3}$’s outdegree is 2 and $v_{4}$’s outdegree is 4. Thus, we consider $v_{2}$’s *ReputationRank* is $\frac{1}{5}p_{v_{1}}+\frac{1}{2}p(v_{3})+\frac{1}{4}p(v_{4})$.

From Example 3, we know that if the members $v_{1}$, $v_{3}$ and $v_{4}$ have a high *ReputationRank*, so does $v_{2}$.

Therefore, we have *g* formulas such as Eq. (1), and we have a system of *g* linear equations. If we compute the social relationship matrix *M*, put the value of the *ReputationRank* into the vector and adopt Katz’s Suppose [19] to normalize the social relationship matrix, the whole formula system could be expressed as

2 $$\begin{aligned} P={M^\text{T}}^{'}P, \end{aligned}$$

where *P* represents the vector consisting of the corresponding *ReputationRank* of each member in the limited state and ${M^\text{T}}^{'}$ denotes the normalized transposed social matrix.

By reorganizing these formulas, we obtain the formula $(I-{M^\text{T}}^{'})P=\mathbf {0}$, where *I* represents a *g*-dimensional unit matrix, and both *P* and $\mathbf {0}$ represent vectors with the length of *g*. The corresponding component of eigenvector *P* whose eigenvalue is 1 represents the *ReputationRank* of the members [12].

### The property of ReputationRank

It should be noticed that a point’s *ReputationRank* is partially consistent with its *Influence*. However, this property alone cannot show the difference between the top and the next. Actually, the *Activeness* also affects the *ReputationRank*.

#### *Example 4*

Given seven members in the SN, as shown in Fig. 1, its corresponding social relationship matrix *M* and its normalized transposed matrix ${M^\text{T}}^{'}$ are as follows:

3 $$\begin{aligned} \begin{array}{cc} M=\left[ \begin{array}{ccccccc} 0 &{} 1 &{} 1 &{} 1 &{} 1 &{} 0 &{} 1\\ 1 &{} 0 &{} 0 &{} 0 &{} 0 &{} 0 &{} 0\\ 1 &{} 1 &{} 0 &{} 0 &{} 0 &{} 0 &{} 0\\ 1 &{} 1 &{} 1 &{} 0 &{} 1 &{} 0 &{} 0\\ 1 &{} 0 &{} 1 &{} 1 &{} 0 &{} 1 &{} 0\\ 1 &{} 0 &{} 0 &{} 0 &{} 1 &{} 0 &{} 0\\ 0 &{} 0 &{} 0 &{} 0 &{} 1 &{} 0 &{} 0 \end{array} \right] , &{} {M^T}^{'}=\left[ \begin{array}{ccccccc} 0 &{} 1 &{} \frac{1}{2} &{} \frac{1}{4} &{} \frac{1}{4} &{} \frac{1}{2} &{} 0\\ \frac{1}{5} &{} 0 &{} \frac{1}{2} &{} \frac{1}{4} &{} 0 &{} 0 &{} 0\\ \frac{1}{5} &{} 0 &{} 0 &{} \frac{1}{4} &{} \frac{1}{4} &{} 0 &{} 0\\ \frac{1}{5} &{} 0 &{} 0 &{} 0 &{} \frac{1}{4} &{} 0 &{} 0\\ \frac{1}{5} &{} 0 &{} 0 &{} \frac{1}{4} &{} 0 &{} \frac{1}{2} &{} 1\\ 0 &{} 0 &{} 0 &{} 0 &{} \frac{1}{4} &{} 0 &{} 0\\ \frac{1}{5} &{} 0 &{} 0 &{} 0 &{} 0 &{} 0 &{} 0 \end{array} \right] \end{array}. \end{aligned}$$

Then we obtain the eigenvector $\alpha = (0.304,0.166,0141,0.105,0.179,0.045,0.061)^\text{T}$ of ${M^T}^{'}$ when the eigenvalue is 1. We can conclude that the *ReputationRank* of each member is almost consistent with their value of *Influence*. It is obvious that the one whose ID is 1 has the highest *ReputationRank* almost for one third of all. We think it is because that Member 1 gains all the reputation from Member 2 who has high *ReputationRank*. What is more, Member 1 has the highest *Influence* and *Activeness*, thus we consider Member 1 is the most popular one in the SN. On the other hand, we find that although Member 2 and Member 3 have the same *Influence*, Member 2’s *ReputationRank* is larger than that of Member 3. The reason is that Member 2 owns one second of Member 3’s *ReputationRank* but Member 3 only owns one fourth of Member 5’ *ReputationRank*. Therefore, we conclude that the *ReputationRank* of a member in an SN is not only related to the *Influence* but also to the *ReputationRank* of their followers and their followers’ *Activeness*.

## Prediction of promoting members in SNs

### Problem statement

The problem we study in this paper is to locate the most “potential” member(s) for promotion by means of elevating it (them) into the skyline. Suppose we have two datasets $D_{1}$ and $D_{2}$. $D_{1}$ represents some data a few years ago and the $D_{2}$ represents that of the following years. If $S_{1}=SKY(D_{1})$, $S_{1}'=SKY(D_{1}-S_{1})$, $S_{2}=SKY(D_{2})$, where the *SKY*() represents the skyline set of the dataset, then $S_{1}'$ is the candidate set in our algorithm. After promoting towards each point in $S_{1}'$, if there exist some points in $S_{1}'$ appearing in $S_{2}$, the prediction is successful. Otherwise, it fails. Since the non-skyline members are candidates for promotion, if a non-skyline member is promoted, some edges are added to the network and the cost of this promotion is to sum up all the costs of the added edges. In addition, we know that added edges may have effects on the metrics of all members in the SN which may need to be recalculated frequently, thus the time cost to do promotion is extremely high. Therefore, finding the suitable non-skyline members promoted to be skyline members with minimum cost is the goal of member promotion in SNs.

### The sort-projection operation

We project all the members into a two-dimensional Cartesian coordinate system in that we only consider the change of *Influence* and *Activeness*, where the *x*-axis represents the *Influence* and the *y*-axis represents the *Activeness*. Taking the candidate *c* as an example, suppose that *c* is dominated by *t* skyline points, it is worth noting that the candidate *c* is dominated in three dimensions (the *Influence* dimension, *Activeness* dimension and *ReputationRank* dimension). But in the process of edge addition, we just consider the dominance on the *Influence* and *Activeness*. Because it is obvious that if a member is not strictly dominated on two dimensions, s/he will not be dominated on three dimensions either [10]. We simply sort the skyline points in ascending order on *x*-axis. What is more, we assume the weights to be arbitrary positive integer numbers from 1 to 10. Some terms mentioned above are defined as follows.

#### **Definition 11**

(*Strictly dominate*) Given an SN *G*(*V*, *E*, *W*), if $p_{1} \prec p_{2}$ and $p_{1}$ is larger than $p_{2}$ on each dimension, we say $p_{1}$ strictly dominates $p_{2}$, denoted by $p_{1}\prec \prec p_{2}$.

#### **Definition 12**

(*Skyline distance*) Given a set *DS* of points in a two-dimensional space, a candidate *c*, and a path *Path*(., .), the skyline distance of *c* is the minimum value of $Path(c, c')$, where $c'$ is a position in the two-dimensional space such that $x.c' \ge x.c$, and $y.c' \ge y.c$, and $c'$ is not strictly dominated by any point in *DS*. We denote the skyline distance as *SkyDist*().

Suppose that *c* is strictly dominated by *t* skyline points in *SKY*(*DS*). For any position $c'$ which is not strictly dominated by any point in *DS* satisfies $x.c' \ge x.c$, and $y.c' \ge y.c$, the promotion from *c* to $c'$ can be viewed as a path from *c* to $c'$, which always goes up along axes. Since we use linear cost functions $cost(c, c')$ as the sum of the weighted length of the segments on the path. We aim to find a path with the minimum value so that the end point $c'$ is not strictly dominated by any skyline point, and $x.c' \ge x.c, y.c' \ge y.c$.

#### **Definition 13**

(*Skyline boundary*) Given a set *SKY* of skyline points in *DS*, we say a point *p* is on the skyline boundary if there exists a point $u \in SKY$ such that $u\prec p$ and there does not exist a point $u' \in SKY$, such that $u' < < p$.

From the definition of skyline boundary, we conclude that the skyline distance of each point on the skyline boundary is 0 [20].

**Fig. 2:**
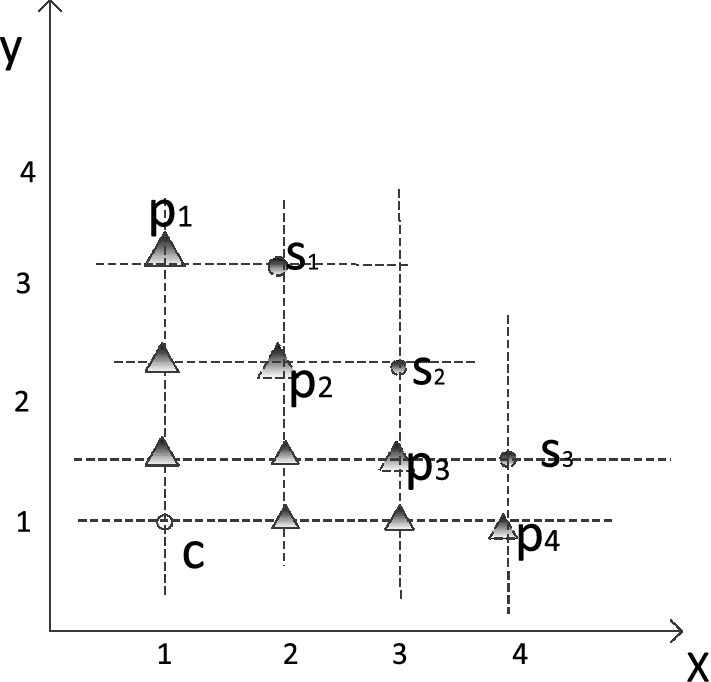
A skyline distance example

Given a candidate *c* and the *t* skyline points $s_{1}$, $s_{2}, \ldots , s_{t}$, we plot the lines $x=x_{c}$, $x=x_{s_{i}}$, $y=y_{c}$ and $y=y_{s_{i}}$, respectively, as shown in Fig. 2, we find there would be some intersections, we use triangles to represent these intersections. We call those intersections on the skyline boundary local optimal points. In Fig.2, $p_{1}$, $p_{2}$, $p_{3}$, and $p_{4}$ are the local optimal points.

Therefore, in the wo-dimensional space, for the candidate *c* and the *t* skyline points $s_{1}$, $s_{2}, \ldots ,s_{t}$, if we have $x.s_{1}< x.s_{2}<\cdots <x.s_{t}$. Without loss of generality, we know $y.s_{1}> y.s_{2}>\cdots >y.s_{t}$. We can conclude that there are $t+1$ local optimal points and the *i*th one $p_{i}$ is given by the following formula:

4 $$\begin{aligned} P_{i}={\left\{ \begin{array}{ll} (x.c, y.s_{1}), \qquad i=1; \\ (x.s_{i-1}, y.s_{i}), \quad 2\le i \le t;\\ (x.s_{t}, y.c), \qquad i=t+1. \end{array}\right. } \end{aligned}$$

Consider a candidate *c* dominated by *t* skyline points $s_{1}$, $s_{2},\ldots , s_{t}$. Let $p_{1}, \ldots , p_{r}$ be the *r* local optimal points determined by *c* and $s_{1}$, $s_{2}, \ldots , s_{t}$, then the skyline distance of *c* is the minimum path from *c* to $p_{i}$.

#### *Example 5*

There is a candidate *c* and $s_{1}, s_{2}, s_{3}$ are skyline points which dominate *c*, as shown in Fig. 2, we can obtain the four local optimal points $p_{1}$, $p_{2}$, $p_{3}$ and $p_{4}$ by Eq. (4), by comparing the path between *c* and $p_{i}$, we can get the skyline distance of *c*. In Fig. 2, the path between *c* and $p_{1}$, $p_{2}$, $p_{3}$, and $p_{4}$ is 2, 2, 2.5 and 3, respectively. Therefore, the skyline distance of *c* is 2.

Algorithm 1 gives the pseudo-codes of the sort-projection operation. Assume that the number of input skyline points is *m*, it is easy to know that the cost of the sorting step is $O(m\log m)$. Then the time cost of remaining step for obtaining the skyline distance mainly depends on the number of local optimal points. From Eq. (4), we know that the time complexity of calculating the local optimal points is *O*(1). Assume that the number of the local optimal points is *k*, then it is easy to know that the time complexity of obtaining the minimum path from candidate *c* to local optimal points is *O*(*k*). Therefore, the time complexity of Algorithm 1 is $O(m \log m+1+k)=O(m \log m)$.

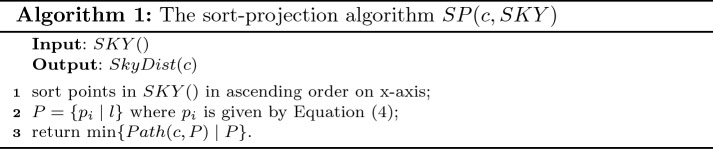



### Pruning by cost and dominance

#### **Definition 14**

(*Promotion plan*) Given an SN *G*(*V*, *E*, *W*), for a candidate $c \in$ candidate set, the promotion plan of *c* includes all the added edges in the process of a promotion attempt.

After obtaining the skyline distance of a candidate, we get the necessary condition for the candidate not being dominated by skyline points. Taking the candidate *c* as an example, assume that $c'$ is the end point after promotion with the skyline distance of *c*, then there exists three different solutions towards the different values of $c'$:

1. If $$x_{c'}=x_{c}$$, then $$x_{c''}=x_{c'}, y_{c''}=y_{c'}+1$$;
2. If $$y_{c'}=y_{c}$$, then $$x_{c''}=x_{c'}+1, y_{c''}=y_{c'}$$;
3. If $x_{c'} \ne x_{c}$ and $y_{c'} \ne y_{c}$, then $x_{c''}=x_{c'}+1, y_{c''}=y_{c'}+1$.

We denote the transformed $c'$ as $c''$. It is obvious that $c''$ could not be dominated by any point at all. If we call the position where a candidate will not be dominated as *GoodPosition*(), we say $c''\in GoodPosition()$. Besides $c''$, all points in the skyline set will not be dominated either. Thus, the dominator set of *c* belongs to *GoodPosition*(*c*).

In view of unequal costs for establishing different edges, it probably takes different costs to promote *c* by different plans. Therefore, we organize all the edges which can be added to the plans against each candidate *c*, respectively, denoted as $E_{c}$ and sort the edges in ascending order of weights. Then we can locate the promotion plans which satisfy the constraints of *GoodPosition*(*c*) from the head of $E_{c}$ and treat them as our original plans. These original plans will be put into a priority queue. When the plan is extracted from the priority queue to be verified, we first of all generate its successive plans and put the successive plans into the priority queue. The successive plans are generated by the Observation 1. Once the plan is verified to be successful to promote the candidates, the process of promotion will be ended. However, if a plan cannot successfully promote the candidates, we can generate some prunable plans based on the failed plan. The guidelines are shown in Observation 2. The idea is the same as the IDP algorithm [3].

#### Observation 1

The successive plans are generated by the following rules:

- If the current plan does not contain the minimum-cost edge $e_{0}$, add it to the current plan.
- If the current plan does not contain any successive edge of $e_{i}$, namely $e_{i+1}$, replace $e_{i}$ with $e_{i+1}$.

#### Observation 2

The prunable plans are generated by the following rules:

#### **Theorem 1**

*If the added edge e connecting node* $v_{i}$* and the candidate node* *c still cannot promote* *c to the skyline set, all the attempts of adding an edge* $e'$* connecting the node* $v_{j}$* and* *c with the same direction as* *e cannot promote* *c to the skyline set either, where* $v_{j} \in \delta (v_{i})$.

#### *Proof*

Assuming that after adding an edge *e*, $v_{i}(I(v), A(v))$ will change to $v_{i}(I'(v), A'(v))$, and *c*(*I*(*c*), *A*(*c*)) will change to $c(I'(c), A'(c))$. Assume there is a point *p* still dominates *c*, if we add an edge $e'$ connecting node $v_{j}$ and *c* with the same direction as *e*, and $v_{j}$ should belong to $\delta (v)$, we consider there should be two situations for $v_{j}$:

1. $v_{j} \ne p$. If $v_{j}$ is a dominator of $v_{i}$ but not be *p*, after adding an edge from $v_{j}$ to *c*, $(I(v_{j}), A(v_{j}))$ will change to $(I'(v_{j}), A'(v_{j}))$, and (*I*(*c*), *A*(*c*)) will change to $(I'(c), A'(c))$, then *p* will still dominate *c*;
2. $v_{j} = p$. If $v_{j}$ is a dominator of $v_{i}$ and dominates *c* when (*I*(*c*), *A*(*c*)) changes to $(I'(c), A'(c))$, after adding an edge from *p* to *c*, (*I*(*p*), *A*(*p*)) will change in $(I'(p), A'(p))$, and (*I*(*c*), *A*(*c*)) will change to $(I'(c), A'(c))$, it is obvious that the changed *p* will still dominate *c* because it dominates *c* before one of the two values corresponding to the metrics increases.

In summary, all the attempts of adding an edge $e'$ connecting the node $v_{j}$ and *c* with the same direction as *e* cannot promote *c* to the skyline set either, where $v_{j} \in \delta (v_{i})$. $\square$

#### **Corollary 1**

*If a promotion plan* $p(e_{1}, \ldots , e_{w})$ *cannot successfully promote its target candidate* *c* *to the skyline set, all the plans with* *w* *edges which belong to* $\prod _{i=1}^{w}{l_{i}}$ *can be skipped in the subsequent verification process against* *c*, *where for each* $e_{i}$ *connecting* $v_{i}$ *and* *c*, $l_{i}$ *is a list containing all the non-existing edges each of which links one member of* $\delta (v_{i})$ *and* *c* *with the same direction as* $e_{i}$ ($i=1, 2, \ldots , w)$, $\prod _{i=1}^{w}{l_{i}}$ *is the Cartesian product of* $l_{i}$.

#### *Proof*

According to Theorem 1, if each edge in $l_{i}$ cannot successfully promote *c*, it means $l_{i}$ cannot do it either. Thus, all the plans with *w* edges belonging to the Cartesian product of $l_{i}$ will fail to promote the candidate.

The steps for pruning some plans are shown in Algorithm 2. Note that $e_{ic}$ denotes the edge which connects from $v_{i}$ to *c*. In Algorithm 2, Lines 3–6 and 7–9 are based on Theorem 1 and Corollary 1, respectively. Thus, we obtain the prunable plans of a given candidate.

Assume that for the candidate *c*, the number of available edges is *k*. For the worst case that all edges belong to available edge set fail to make *c* successfully promoted, suppose that the number of nodes which dominate *c* is *h*, then the time complexity of generating some prunable edges against each failed point is *O*(*hk*). Furthermore, the time complexity of generating the prunable plans is *O*(1). Thus, the total time complexity in the worst case is *O*(*hk*). $\square$

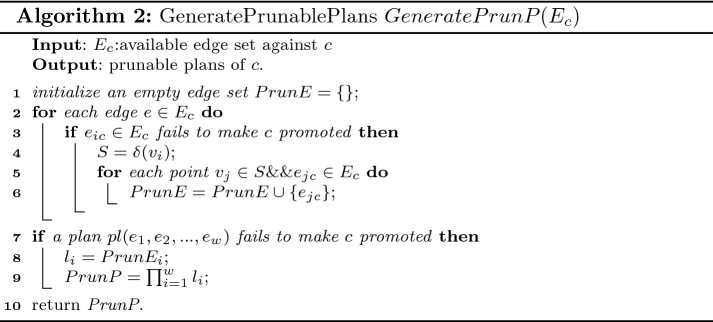



### Verification of the result

After pruning some meaningless plans based on promotion cost and dominance, the remaining plans will be carried out for promotion. It is well known that the skyline set may change after a promotion attempt, thus the candidate may still be dominated by other members. Therefore, the final verification must be executed to examine the results of the promotions.

It is time-consuming if we recalculate the skyline set after each promotion. We notice that those points which do not dominate the candidate before promotion would not dominate it after promotion either. Thus we can ignore it in the verification process. Therefore, after pruning, we should just consider the following situations when verifying:

- The points which dominate the candidate before promotion.
- The points which are contained in the promotion plans.

### The PromSky algorithm

The whole process of member promotion in an SN is presented in Algorithm 3. Line 2 represents the generation of candidate set. Line 4 represents a preprocessing phase by generating the sorted available edges. The skyline distance of each candidate is calculated in Line 5. Then *GoodPosition*() is generated in Lines 6–14. The point $c'$ is the promoted point with the skyline distance of *c*. Line 16 shows that the corresponding promotion plans are generated and put into the priority queue *Q*. Once the queue is not empty, we fetch the plan with minimum cost for further verification. Line 18 shows that before verifying the plan, we first generate its children plans by Observation 1 so that we can verify all the possible plans in ascending order of cost. Lines 21–24 represent that after checking based on the result verification strategy the result will be output if the promotion succeeds. If not, some prunable plans will be generated. The generation of prunable plans are showed in Line 28. Lines 25–26 represent that if the plan is in the prunable list, there is no need of further verification. Lines 19–20 show that after a successful promotion, the process will halt once we encounter a plan with the higher cost.

We estimate the time complexity of our PromSky algorithm in the worst case. Assumed that the candidate set is *M*, it takes *O*(|*M*|) time to build its available edge set and $O(|M|\log |M|)$ time to calculate the skyline distance. For the recursion on the basis of each plan, the worst time complexity of generating the children plans is *O*(|*M*|). It will take $O(\log |M|)$ to build and search the min heap. The generation process of the prunable list will cost $O(|m|^{2})$. We build an index such as $B^{+}$ tree for speeding up the search in the prunable list, whose time cost can maintain steady at around $O(|M|\log |M|)$. The result checking phase will take *O*(|*M*|) at worst. Theoretically, the worst time complexity of Algorithm 3 is $O(|M|^{3})$(However, the algorithm usually reaches the result at early time in experiments).

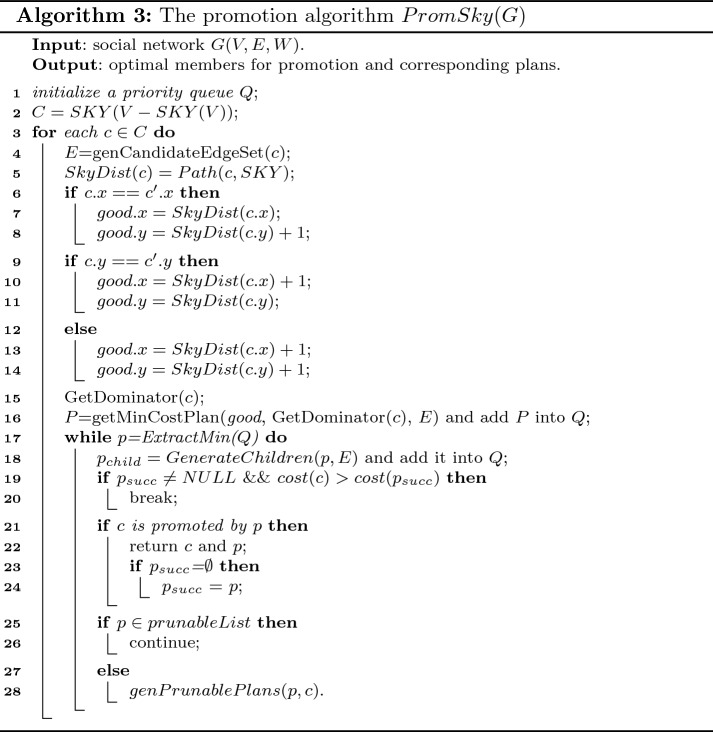



### Analysis

In the SkyBoundary algorithm, Peng et al. [4] only used the *Authoritativeness(indegree)* and *Hubness(outdegree)* as the metrics, and described the plan limitation for promotion by bringing forward a new concept called “promotion boundary”, and then proposed an effective boundary-based pruning strategy to prune the searching space. In this paper, we propose the concept of *ReputationRank* based on the Google’s *pagerank* algorithm and add it as a measure attribute to describe the importance of a member, which helps to improve the accuracy of the prediction to some degree. Then we present the definition of skyline distance to obtain the necessary condition for not being dominated. At the same time, it also helps a lot to cut down the number of promotion plans.

On the other hand, when making a comparison on the time, from the size of the candidate set, when experimenting on the real-world datasets, the candidate set is all the non-skyline set in the SkyBoundary algorithm [4]. However, we carry a skyline query over the non-skyline set under the consideration of three dimensions and take the infra-skyline as the candidates so that remarkably pruning the size of the candidates and controlling the result set in a reliable range. On the other hand, by calculating the skyline distance of the candidate, we obtain the minimum path from the candidate’s position to where not being strictly dominated. Then after trying all the positions belong to *GoodPositions*(), we can get the promotion plans that succeed in promoting the candidate by verifying the plans one by one. However, in [4], the SkyBoundary algorithm although pruned some meaningless plans based on the promotion boundary and got the constraint of promotion plans. They merged all the possible good points with the skyline points which dominate the candidate, then verified it in sequence to get the minimum cost one. Apparently, their method needs more time compared to our proposed algorithm.

## Experimental analysis

### Setup

The experiments are implemented using C++ with Visual Studio 2010 and conducted on an Intel Core CPU i75500U@2.4GHZ machine with 8G RAM and 1 TBytes Hard disk running on Windows 7. We use two datasets for the experiments.

1. WikiVote dataset: Wikipedia is an encyclopedia that any volunteers all over the world are able to write on it collaboratively. The dataset^1^ contains all administrator elections and vote history data from 2004 to 2008. 2794 elections with 103663 total votes and 7066 users participating in the elections are contained in the dataset. Users are those who cast votes or are voted on. Each record includes 5 parts such as *E*, *T*, *U*, *N*, *V*. They correspondingly represent whether the election is successful or not, the time election is closed, user id (and username) of editor that is being considered for promotion, user id (and username) of the nominator and each voter’s voting results. Nodes in the network represent users and a directed edge from node *p* to node *q* represents that user *p* votes on user *q*. We set all the weights to be random integers between 1 and 10 for simplicity.
2. DBLP dataset: DBLP^2^ is a computer science bibliography website. Each record of the DBLP dataset consists of authors’ names, paper title and published year. We collect all the records from 1992 to 2016. For a paper that was accomplished by several authors, we think the first author generally makes major contributions and the others do minor contributions. Thus, we build a directed graph by the co-author network. Nodes in the graph represent the authors and the directed edges with the first author as the end node and the other authors, respectively, as the start nodes represent the relationships between authors. We set all the weights of edges to be random integers between 1 and 10 for simplicity.

### Results

RanSky algorithm: we pick up a candidate from the candidate set, and we randomly choose some added edges from the available edges until this candidate being successfully promoted. We denote it as a RanSky algorithm which is an adaptive version of the random algorithm in [4].

#### Promotion cost comparisons

In this set of experiments, we make a comparison on promotion costs of our PromSky algorithm with the RanSky algorithm. We consider the sum of the added edges’ weights as the promotion cost of the Random algorithm. Then we use the PromSky algorithm to find out the optimal promotion plans and calculate their promotion costs, respectively.

**Fig. 3:**
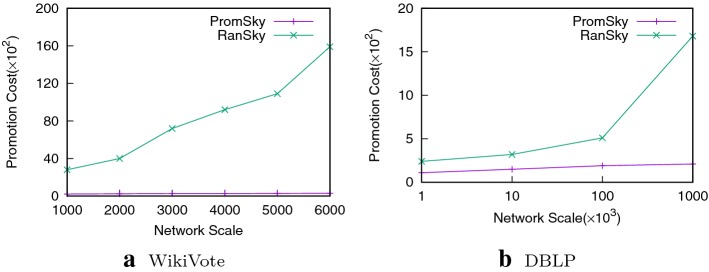
Promotion cost comparison with the Random algorithm

Figure 3 illustrates the promotion costs of the two algorithms on WikiVote and DBLP datasets, respectively. The promotion costs of the two algorithms both grow with the increase of the network scales. It is obvious that the promotion cost of RanSky algorithm is much more than the PromSky algorithm, which means that our PromSky algorithm always provides the optimal plans. What is more, the differences between the two promotion costs in both datasets basically grow along with the scale of the network. By the way, we think the promotion cost on the WikiVote dataset is much more than the cost on the DBLP dataset is due to the existing connected edges on the WikiVote are less than that on the DBLP dataset.

#### Successful rate comparisons

**Fig. 4:**
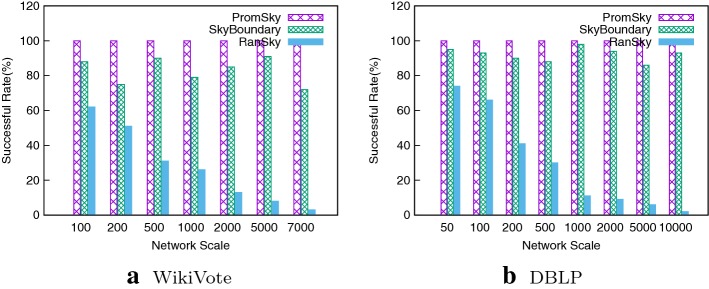
Successful rate comparison on various network scales

We make a comparison of our PromSky algorithm with the SkyBoundary algorithm and RanSky algorithm in various network scales. The target candidate is the one who can be successful promoted randomly selected from the result of our PromSky algorithm and its promotion cost is the optimal cost. We add *e* edges picked from the available edges against the candidate according to the PromSky and SkyBoundary algorithm, respectively, and add *e* edges randomly picked from the available edges, then we verify the result. We calculate the promotion successful rate by counting the number of successful promotions in ten times promotion attempts. We conduct the experiments on both WikiVote and DBLP. From Fig. 4, we find that the SkyBoundary algorithm and the RanSky algorithm cannot guarantee the promotion’s success even though we picked the optimal candidate and achieved the minimal promotion cost, the RanSky algorithm works worse especially. On the contrary, our PromSky algorithm performs well in various network scales. This is because we add more attributes in our PromSky algorithm for a member that it should increase the number of skyline set. Thus our successful promotion rate is higher in various network scales.

#### Prediction on DBLP

In this section, we record the predicted potential stars and the skyline authors detected by our algorithm from 1992 to 2016. For each year’s data, we consequently combine the current yearly data with its previous 4 years’ data to generate a 5-year sub-network because publications too long ago will have little impact on the contributions made by the authors of the time and only one year’s publications cannot accurately reflect the contributions of the authors [4]. Then we run our PromSky algorithm on each sub-network (from 1996 to 2016) to verify the corresponding yearly potential stars and those skyline authors in the following couple of years. The skyline authors are obtained by conducting a skyline query over the *Influence* dimension, *Activeness* dimension and *ReputationRank* dimension. The potential authors are the predicting results of our PromSky algorithm. We can get the successful rate using the number of potential stars promoted into skyline in the next few years divided by the size of the whole potential star set, namely

5 $$\begin{aligned} r={\mathrm{PN}}/{\mathrm{CS}}, \end{aligned}$$

where “*r*” denotes the successful rate, and “PN” and “CS” are the number of successfully promoted members and the number of all the candidates, respectively.

The skyline authors and potential stars for each year are illustrated in Table 1. From Table 1, we can see each year’s skyline authors and potential skyline authors from 1996 to 2016. We think that if the potential skyline author become a skyline author in the next few years, the promotion is successful, otherwise, it fails. We obtain the number of the potential candidates is 20 by merging the duplicated potential stars and removing the potential stars of the year 2016 because it is unable to be verified, and the number of the potential candidates who appear in the next skyline authors is 13. Those names which are in italic represent the successfully promoted candidates. Therefore, we conclude that the successful rate is 65%. However, in the previous research [4], when conducting the experiments on the dataset from 1971 to 2012, we find the successful rate is only 48%. It shows that our algorithm is more accurate than the previous.

**Table 1:** Skyline authors and potential stars from 1996 to 2016

Year	Skyline	Potential skyline
1996	Robert L. Glass, David Wilczynski	Robert W. Floyd
1997	Noga Alon, Jean P, Caxton Foster	Peter Kron
1998	Noga Alon, Robert L. Glass, V. Kevin M	Carl Hewitt, Bill Hancock
1999	*Robert W. Floyd*, Noga Alon, Honien Liu	Paul A.D., Alan G. Merten
2000	*Bill Hancock, Peter Kron*	Paul A.D.
2001	*Bill Hancock*, Nan C. Shu	Pankaj K. Agarwal
2002	*Bill Hancock*, Charles W. Bachman, Daniel L. Weller	Pankaj K. Agarwal
2003	Bill Hancock, Daniel L. Weller	Elisa Bertino, Alan G. Merten
2004	*Pankaj K. Agarwal*, Morton M. Astrahan, David R. Warn	Elisa Bertino, Mary Zosel
2005	Gary A. Kildall, Diane Crawford, Hans-Peter Seidel, *Erik D.Demaine*	Carl Hewitt
2006	Noga Alon, Diane Crawford, *Pankaj K. Agarwal*	Ingo H. Karlowsky, Louis Nolin
2007	*Elisa Bertino*, G. RuggiuW, J. Waghorn, M.H. Kay, Erik D. Demaine	T. William Olle
2008	Diane Crawford, *Paul A.D.*	B.M. Fossum
2009	Wen Gao, Xin Li, Jun Wang, P.A. Dearnley, Giampio Bracchi, Paolo Paolini, Ajith Abraham	H. Schenk, Gordon E. Sayre
2010	Xin Li, *B.M. Fossum*, J.K. Iliffe, Wen Gao, *Mary Zosel*, Wei Wang	Paul Mies, Ingo H. Karlowsky
2011	Xin Li, Gordon E. Sayre, T. William Olle	Peter Sandner
2012	H. Vincent Poor, *Peter Sandner*, Ulrich Licht	Yan Zhang
2013	*Ingo H. Karlowsky*, Heidi Anlauff, Günther Zeisel	Guy G. Boulaye
2014	*Yan Zhang*, Yu Zhang, Gordon E. Sayre, Witold Pedrycz	Carl Hewitt
2015	Harold Joseph Highland, Bernard Chazelle	Won Kim
2016	*Won Kim*, Dale E. Jordan, *B.M. Fossum*	Nan C. Shu

#### Time cost comparisons

We conduct the experiments to compare the time costs of our PromSky algorithm with the SkyBoundary algorithm on two datasets. For the reason of intolerable time complexity, we do not take the RanSky algorithm to be a compared algorithm.

**Fig. 5:**
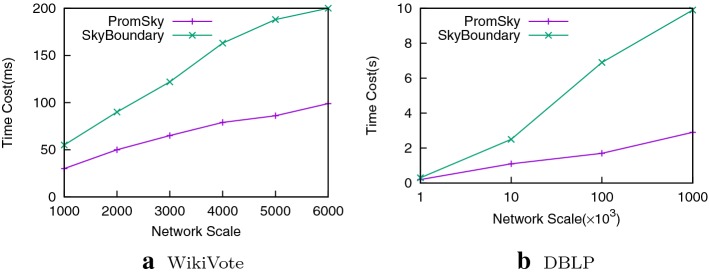
Time cost comparison on various network scales

Figure 5 shows the average running time under different network scales. From Fig. 5, we can see that as the network scale grows, the running time also increases and our PromSky algorithm is faster than the SkyBoundary algorithm whatever the network scale is. This is because the candidates in SkyBoundary algorithm are all the non-skyline set but we carry the skyline query over the non-skyline set and take the infra-skyline as the candidates thus remarkably reducing the size of the candidates and controlling the result in a reliable range to a great extent. Besides, by bringing forward the skyline distance, we can reduce the searching space of promotion plans remarkably.

## Conclusions

In this paper, we propose an improved member promotion algorithm in SNs, which aims at discovering the most potential stars which can be promoted into the skyline with the minimum cost. By adding the attribute of *ReputationRank*, we describe members’ importance more precisely. Then we introduce the skyline distance to prune the data points for not being dominated. At the same time, it also helps a lot to reduce the number of promotion plans. Experimental results on the DBLP and WikiVote datasets illustrate the effectiveness and efficiency of our approach.

## Footnotes

1 http://snap.stanford.edu/data/wiki-Vote.html.

2 http://dblp.org/.
